# Systemic therapy for hepatocellular carcinoma, from the early to the advanced stage: a Japanese perspective

**DOI:** 10.1093/jjco/hyaf017

**Published:** 2025-02-03

**Authors:** Masafumi Ikeda, Chigusa Morizane, Makoto Ueno, Takuji Okusaka, Hiroshi Ishii, Junji Furuse

**Affiliations:** Department of Hepatobiliary and Pancreatic Oncology, National Cancer Center Hospital East, 6-5-1 Kashiwanoha, Kashiwa, Chiba 277-8577, Japan; Department of Hepatobiliary and Pancreatic Oncology, National Cancer Center Hospital, 5-1-1 Tsukiji, Chuo-ku, Tokyo 104-0045, Japan; Department of Gastroenterology, Kanagawa Cancer Center, 2-3-2, Nakao 2-chome, Asahi-ku, Yokohama 241-8515, Japan; Department of Hepatobiliary and Pancreatic Oncology, National Cancer Center Hospital, 5-1-1 Tsukiji, Chuo-ku, Tokyo 104-0045, Japan; Gastrointestinal Medical Oncology, Chiba Cancer Center, 666-2 Nitona-cho, Chuo-ku, Chiba 260-8717, Japan; Department of Hepatobiliary and Pancreatic Oncology, National Cancer Center Hospital, 5-1-1 Tsukiji, Chuo-ku, Tokyo 104-0045, Japan

**Keywords:** hepatocellular carcinoma, systemic therapy, molecular-targeted therapy, immunotherapy, early stage, intermediate stage, advanced stage

## Abstract

Systemic therapy has now become mainstream for the treatment of hepatocellular carcinoma (HCC) and is also changing from molecular-targeted therapy, such as with sorafenib and lenvatinib, to immunotherapy, such as with the atezolizumab plus bevacizumab and durvalumab plus tremelimumab combination regimens. Molecular-targeted therapy is selected as the first-line treatment when immunotherapy is not indicated or as second- or later-line treatment when immunotherapy is ineffective. It is necessary to select the appropriate treatment taking into consideration the expected treatment efficacy and adverse events, as well as the hepatic reserve. Currently, newer agents and combination regimens as first-line/second-line treatment for advanced-stage HCC, combined therapy with transarterial chemoembolization for intermediate-stage HCC, and perioperative adjuvant therapy for curative treatment for early-stage HCC are being developed. Therefore, systemic therapy is now indicated for any stage of the disease. While local therapies were previously used as the main treatment strategy for HCC, systemic therapy in combination with local therapies is being actively attempted at present. Systemic therapy is currently the main focus of development of novel treatments for HCC.

## Teaser

Systemic therapy is currently the mainstream treatment for hepatocellular carcinoma. Its application is expanding from the intermediate stage to the early stage of the disease, and novel treatments are being actively developed.

## Introduction

**Figure 1:**
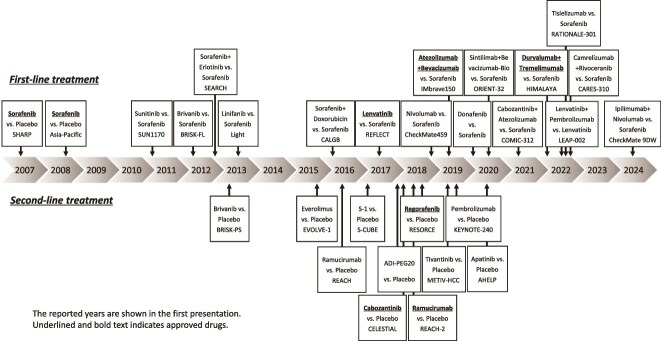
Classification of the drugs available currently for the treatment of unresectable hepatocellular carcinoma.

**Figure 2:**
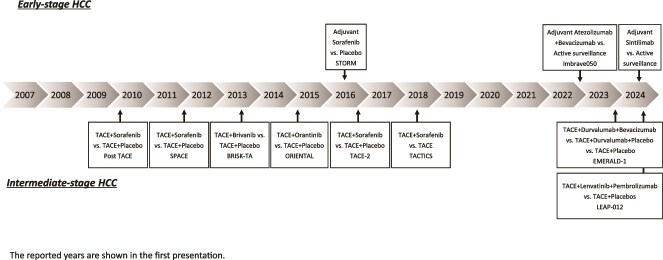
Treatment strategy for intermediate-stage hepatocellular carcinoma.

Effective systemic therapy for hepatocellular carcinoma (HCC) began with the development of sorafenib (Sora), a multi-kinase inhibitor [1,2]. More recently, effective combined immunotherapy regimens have been introduced, such as atezolizumab (Atezo) plus bevacizumab (Bev) [3] and durvalumab (Durva) plus tremelimumab (Treme) [4], which are the mainstays of systemic therapy at present. Other agents/regimens available for use include lenvatinib (Len) [5] and Durva [4] as first-line therapy, and regorafenib [6], ramucirumab [7], and cabozantinib [8] as second- or later-line therapy, bringing the total number of systemic therapeutic agents now available in Japan to treat HCC to eight agents (Fig. 1). Furthermore, even though systemic therapy has been mainly used for patients with advanced-stage disease as classified according to the Barcelona Clinic Liver Cancer (BCLC) group staging system [9], the indications have now been expanded to include both intermediate- and early-stage HCC (Table 1; Fig. 2). In this article, we outline the current status and ongoing clinical trials of systemic therapies for advanced-stage, intermediate-stage, and early-stage HCC, as classified according to the BCLC staging system.

**Table 1:** Treatment strategies for hepatocellular carcinoma

	**Early stage**	**Intermediate stage**	**Advanced stage**
Patients	Single tumor nodule >2 cm or ≤3 nodules measuring ≤3 cm PS 0 Child–Pugh A–B	Multinodular HCC with no vascular invasion or extrahepatic spread PS 0 Child–Pugh A–B	Vascular invasion Extrahepatic metastasis PS 1–2 Child–Pugh A–B
Standard treatment	Hepatectomy Liver transplantation Ablative therapy (RFA, MWA, ethanol injection, etc.)	TACE	Systemic therapy (first-line): Atezolizumab + bevacizumab Durvalumab + tremelimumab Sorafenib Lenvatinib Durvalumab Systemic therapy (second-line or later): Regorafenib Ramucirumab (AFP ≥ 400 ng/mL) Cabozantinib
Development status of systemic therapy	Perioperative treatment: neoadjuvant treatment and adjuvant treatment	TACE + systemic therapy Systemic therapy	Novel immunotherapy Novel molecular-targeted therapy Novel combined therapy

PS, performance status; RFA, radiofrequency ablation; MWA, microwave ablation; TACE, transarterial chemoembolization.

## Systemic therapy for advanced-stage HCC

### First-line treatment

At present, use of Sora [1,2], Len [5], Atezo + Bev [3], Durva + Treme [4], and Durva [4] for the treatment of HCC is reimbursed by the National Health Insurance program, and the agents are available for routine use in Japan (Fig. 1). Among these, Atezo + Bev [3] and Durva + Treme [4], which have been shown to provide significantly greater survival benefit than Sora, are the most popular regimens at present. Sora, Len, or Durva is selected for patients who are not suitable candidates to receive Atezo + Bev and Durva + Treme as first-line therapy.

### Atezo + Bev

Atezo + Bev is a combined immunotherapy regimen consisting of Atezo, an anti-PD-L1 antibody, and Bev, an anti-VEGF-A antibody. PD-L1 is an immunosuppressive molecule that inhibits T-cell activation by binding with PD-1 expressed on the T cells. Bev improves the immunosuppressive tumor microenvironment caused by Vascular Endothelial Growth Factor (VEGF)-induced inhibition of dendritic cell maturation and tumor angiogenesis by promoting infiltration of the tumor by activated CD8-positive T cells. Bev can ameliorate and potentiate the effects of Atezo by inhibiting the maturation of dendritic cells and inhibiting tumor angiogenesis [3]. It is also expected to exert direct antitumor effects via inhibiting tumor angiogenesis [10]. Atezo + Bev was demonstrated in a phase III study (IMbrave150) [3] to yield significantly better overall survival, progression-free survival, and objective response rates in patients with HCC as compared with Sora (Table 2). The most commonly reported adverse events are hypertension, proteinuria, fatigue, increased AST, and pruritus; in addition, some serious immune-related adverse events and bleeding events have also been reported.

**Table 2:** Comparison of the patient backgrounds, treatment efficacy, and adverse events between the IMbrave150 and HIMALAYA trials

	**IMbrave150**		**HIMALAYA**	
	**Atezolizumab + bevacizumab**	**Sorafenib**	**Durvalumab + tremelimumab**	**Sorafenib**
Number of cases enrolled	336	165	393	389
BCLC stage B, %	15	15	20	17
BCLC stage C, %	82	81	80	83
Etiology: hepatitis B, %	49	46	31	31
Etiology: hepatitis C, %	21	22	28	27
Etiology: nonviral, %	30	32	41	43
Child–Pugh A5, %	72	73	75.1	71.2
Child–Pugh A6, %	28	27	23.4	26.2
Vascular invasion, %	38	43	26.2	25.7
Extrahepatic spread, %	63	56	53.2	52.2
Follow-up period (median), months	15.6	15.6	33.18	32.23
Overall survival (median), months	19.2 (17.0–23.7)	13.4 (11.4–16.9)	16.4 (14.2–19.6)	13.8 (12.3–16.1)
Hazard ratio (95% CI)	0.66 (0.52–0.85)		0.78 (0.65–0.92)	
Progression-free survival (median), months	6.9 (5.7–8.6)	4.3 (4.0–5.6)	3.78 (3.68–5.32)	4.07 (3.75–5.49)
Hazard ratio (95% CI)	0.65 (0.53–0.81)		0.90 (0.77–1.05)	
Objective response rate (RECIST v1.1), %	30	11	20.1	5.1
Complete response, *n* (%)	25 (8)	1 (< 1)	12 (3.1)	0
Partial response, *n* (%)	72 (22)	17 (11)	67 (17.0)	20 (5.1)
Stable disease, *n* (%)	144 (44)	69 (43)	157 (39.9)	216 (55.5)
Progressive disease, *n* (%)	63 (19)	40 (25)	157 (39.9)	153 (39.3)
Disease control rate, %	74	55	60.1	60.7
All-grade immune-mediated AEs, %	ND	ND	35.8	8
Grade 3–4 immune-mediated AEs, %	ND	ND	12.6	2.4
Immune-mediated AEs necessitating use of steroid therapy, %	12.2	ND	20.1	1.9
All-grade bleeding-related AEs, %	25.2	17.3	ND	ND
Grade 3–4 bleeding-related AEs, %	6.4	5.8	0.5	1.1

ND, no data; CI, confidence interval; AE, adverse event.

### Durva + Treme

Durva + Treme is a combined immunotherapeutic regimen consisting of Durva, an anti-PD-L1 antibody, and Treme, an anti-CTLA-4 antibody. Durva is a human monoclonal antibody that binds to and inhibits human PD-L1, thereby suppressing tumor immune escape mechanisms and triggering an antitumor immune response. Treme targets cytotoxic T-lymphocyte antigen 4 (CTLA-4) and activates T cells by blocking the actions of CTLA-4, thereby enhancing the immune responses against cancer cells and inducing cancer cell death [4]. A distinctive feature of this regimen is that Treme is administered as a single high dose.

In a phase III (HIMALAYA) study [4] conducted to compare Durva + Treme or Durva alone with Sora alone as primary treatment in patients with unresectable HCC (Table 2), Durva + Treme was demonstrated to be superior to Sora in that it yielded significantly better survival. Furthermore, Durva alone was shown to be noninferior to Sora in terms of the overall survival. In particular, improved 3- and 4-year survival rates are characteristic of the long-tail effect or tail plateau. Recently, favorable results of Durva + Treme after 5 years of follow-up were reported, with a 5-year survival rate being 19.6% [11]. While the progression-free survivals were not significantly different among Sora, Durva + Treme, and Durva, the objective response rate to Durva + Treme was superior as compared with that to Sora. In terms of the adverse events, 20.1% of patients treated with Durva + Treme experienced immune-related adverse events requiring steroid therapy, but there were no cases of treatment-related bleeding events. This Durva + Treme regimen is a combined immunotherapy regimen consisting of immune checkpoint inhibitors only; it is the only regimen that does not inhibit angiogenesis, which makes it easier to use it in patients with a high risk of bleeding and proteinuria.

### Atezo + Bev or Durva + Treme?

Both the Atezo + Bev [3] and Durva + Treme [4] regimens have outperformed Sora in terms of the overall survival. So, which of the two regimens could we select for first-line treatment? There are some differences in the eligibility criteria of both clinical trials, treatment outcomes, and adverse events between these two regimens (Table 2). The HIMALAYA trial excluded patients with severe vascular invasion, such as tumor thrombosis in the main portal vein. Atezo + Bev has been demonstrated to yield a better median and hazard ratio of overall survival and progression-free survival, and a more favorable objective response rate as compared with Durva + Treme. In regard to adverse events, immune-related adverse events were more frequent with Durva + Treme, while bleeding-related adverse events, proteinuria, and hypertension were more frequent with Atezo + Bev, suggesting that both regimens have their merits and demerits. Since no randomized controlled trials have been conducted to directly compare the two regimens, either could be recommended. The Japanese Clinical Practice Guidelines for Liver Cancer also revised their treatment algorithm for systemic therapy after the introduction of Durva + Treme, recommending either Atezo + Bev or Durva + Treme as the first-line therapy for patients in whom combined immunotherapy is not contraindicated due to reasons such as the presence of autoimmune disease (Table 3). Thus, no determination of superiority or inferiority has been made yet between the two regimens. In patients who are not suitable candidates to receive combined immunotherapy, Sora, Len, or Durva alone is recommended as first-line therapy. Similar to the Japanese Clinical Practice Guidelines for Liver Cancer, the NCCN Guideline 2024 v1 as well as the BCLC staging system list Atezo + Bev and Durva + Treme as the regimens of first choice.

**Table 3:** Treatment selection of systemic therapy for unresectable hepatocellular carcinoma

**First-line therapy**	**Second- or later-line therapy**
Atezolizumab + bevacizumab	Sorafenib Lenvatinib Regorafenib Ramucirumab Cabozantinib Durvalumab + tremelimumab Durvalumab
Durvalumab + tremelimumab	Sorafenib Lenvatinib Regorafenib Ramucirumab Cabozantinib Atezolizumab + bevacizumab
If neither of the above regimens is indicated	
Sorafenib	Regorafenib Ramucirumab Cabozantinib Lenvatinib
Lenvatinib	Sorafenib Regorafenib Ramucirumab Cabozantinib
Durvalumab	Sorafenib Regorafenib Lenvatinib Ramucirumab Cabozantinib

Underlined drugs are those for which evidence has been obtained from randomized controlled trials.

Recently, the high costs of the drugs place a heavy burden on both the patients and society [12]. The treatment cost for the first cycle of Atezo + Bev treatment (3-week cycles) as of January 2025 is 633 262 Japanese Yen (JPY). The treatment cost for the first cycle of Durva + Treme treatment is 240 817 JPY, and that for each subsequent cycle (4-week cycles) is 305 421 JPY assuming an average weight of the patients of 60 kg, while the cost per first cycle of 4 weeks was 287 422 JPY in lenvatinib and 366 273 JPY in sorafenib. Thus, the cost of combined immunotherapy was high, especially Durva + Treme. Therefore, we should consider the cost of the treatment when selecting the most appropriate treatment.

At present, selection between the two regimens is left to the judgment of the attending physician. How both regimens are being used in clinical practice is currently being investigated in a prospective observational study of systemic therapy for HCC (PRISM: UMIN000040488) and a retrospective study based on the database of the Japan Liver Cancer Study Group (HERITAGE: UMIN000046567); the results of these studies may be expected to clarify the current status of systemic therapy for HCC using these two regimens in Japan. Thus, the advent of Atezo + Bev and Durva + Treme has led to a change in the primary treatment strategy for advanced-stage HCC, with a shift from the era of molecular-targeted therapy to the era of combined immunotherapy.

### Other first-line treatment regimens for which favorable results have been reported

Other agents/regimens for first-line treatment that have shown survival benefit in global pivotal phase III trials include tislelizumab [13], camrelizumab + rivoceranib [14], and nivolumab (Nivo) + ipilimumab (Ipi) [15], and those that have shown survival benefit in China alone include donafenib [16], sintilimab plus bevacizumab-biosimilar [17], and anlotinib plus penpulimab [18]. Only the global trials are discussed here.

### Tislelizumab

Tislelizumab is an anti-PD-1 antibody, and a phase III trial (RATIONALE-301) [13] was conducted to compare this drug with Sora. The primary endpoint was the overall survival. The study failed to demonstrate superiority of tislelizumab over Sora in terms of the overall survival, but did demonstrate noninferiority [median overall survival: tislelizumab 15.9 months vs. Sora 14.1 months; hazard ratio, 0.85; 95% confidence interval (CI), 0.71–1.02]. However, the HIMALAYA trial [4] had also demonstrated the noninferiority of Durva alone to Sora and is already approved as a first-line treatment worldwide, making the future positioning of tislelizumab as first-line treatment uncertain.

### Camrelizumab + rivoceranib

A global phase III trial with China as the major participant compared camrelizumab + rivoceranib with Sora alone (CARES-310) [14]. Significantly better outcomes were reported in the camrelizumab + rivoceranib arm, in terms of both the overall survival (median: camrelizumab + rivoceranib 22.1 months vs. Sora 15.2 months; hazard ratio, 0.62; 95% CI, 0.49–0.80; *P* < .0001) and progression-free survival (median: camrelizumab + rivoceranib 5.6 months vs. Sora 3.7 months; hazard ratio, 0.52; 95% CI, 0.41–0.65; *P* < .0001). This regimen contains a VEGF-inhibiting multi-kinase inhibitor that is not Bev and is an unprecedented combination regimen with a relatively strong therapeutic effect. Approval of this regimen in Japan is also expected after completion of the Japanese bridging study with the CARES-310 trial.

### Nivo + Ipi

In a previous phase II trial (CheckMate 040), favorable response rates and overall survival were reported for Nivo + Ipi, and a global phase III trial was conducted to compare Nivo + Ipi with Sora or Len [15]. The combination regimen of Nivo + Ipi consists of anti-PD-1 antibody plus anti-CTLA-4 antibody, similar to Durva + Treme. The difference between the two regimens is that Treme is administered only in the first cycle, while ipilimumab is administered four times. In addition, the CheckMate-9DW trial included a control group that received Len, whereas the HIMALAYA trial only used Sora as control. The primary endpoint, the median overall survival, was 23.7 months in the Nivo + Ipi arm and 20.6 months in the Sora/Len arm (hazard ratio 0.79; 95% CI, 0.65–0.96; *P* = .018), indicating a significantly better overall survival in the former. The first 12 months were better in the Sora/Len arm, and early deterioration of the survival curve in the Nivo + Ipi arm was also observed, which is often specific to immunotherapy. The objective response rate was also better in the Nivo + Ipi arm (36% in the Nivo + Ipi arm, 13% in the Sora/Len arm). In the Nivo + Ipi arm, grade 3–4 immune-related adverse events were observed in 28%, immune-related adverse events requiring steroid treatment in 29%, and treatment-related death in 4%, raising some concerns.

### Ongoing phase III trials of regimens for first-line treatment

The ongoing phase III trials of regimens for first-line treatment are listed in Table 4. Development of combined triplet regimens containing immune checkpoint inhibitors targeting TIGIT (tiragolumab, IMbrave152) or LAG-3 (relatlimab, RELATIVITY trial) is under way (Table 4). For tiragolumab, a phase Ib/II trial (MORPHEUS-Liver) has been conducted comparing the combination regimens of tiragolumab + Atezo + Bev and Atezo + Bev, and a favorable objective response rate/progression-free survival has been reported for the triplet regimen [19]. As for the development of newer agents, trials of TIM-3 (TSR-022, NCT03680508) and bispecific antibodies (ERY974, NCT05022927) are also underway, providing hope for further improvement in the efficacy of therapeutic regimens for HCC.

**Table 4:** Main ongoing clinical trials for hepatocellular carcinoma

**Trial name**	**Experimental arm**	**Control arm**	**Planned sample size**	**Primary endpoint**	**Trial ID**
**First-line treatments for advanced-stage hepatocellular carcinoma**					
IMPACT	Atezolizumab + bevacizumab + TACE	Atezolizumab + bevacizumab	600	Overall survival, conversion rate	jRCTs051230037
RELATIVITY-106	Relatlimab + nivolumab + bevacizumab	Nivolumab + bevacizumab	162	Objective response rate	NCT05337137
IMbrave152/SKYSCRAPER-14	Tiragolumab + atezolizumab + bevacizumab	Atezolizumab + bevacizumab	650	Progression-free survival/overall survival	NCT05904886
GEMINI	Volrustomig, volrustomig + bevacizumab, volrustomig + lenvatinib		180	Objective response rate, adverse events, progression-free survival	NCT05775159
**Second-line treatments for advanced-stage hepatocellular carcinoma**					
IMbrave251	Atezolizumab + sorafenib or lenvatinib	Sorafenib or lenvatinib	554	Overall survival	NCT04770896
LIVIGNO-1	Livmoniplimab + budigalimab	Sorafenib or lenvatinib	120	Objective response rate	NCT05822752
SELECT-400	Ramucirumab	Lenvatinib	130	Overall survival (noninferiority)	jRCT1031210092
SUCCEED	Sorafenib	Lenvatinib	164	Overall survival	jRCT1031210167
**Intermediate-stage hepatocellular carcinoma**					
EMERALD-3	TACE + durvalumab + tremelimumab	TACE alone	525	Progression-free survival	NCT05301842
	TACE + durvalumab + tremelimumab + lenvatinib				
CheckMate74W	TACE + ipilimumab + nivolumab	TACE + placebo	765	Time to TACE progression/overall survival	NCT04340193
	TACE + nivolumab				
TACE-3	TACE + nivolumab	TACE alone	522	Overall survival/time to TACE progression	NCT04268888
TALENTACE	TACE + atezolizumab + bevacizumab	TACE alone	342	Progression-to-TACE-free survival/overall survival	NCT04712643
ABC-HCC	Atezolizumab + bevacizumab	TACE alone	434	Time to TACE refractoriness/overall survival	NCT04803994
**Early-stage hepatocellular carcinoma**					
EMERALD-2	Durvalumab + bevacizumab	Placebo + placebo	908	Recurrence-free survival	NCT03847428
CheckMate9DX	Nivolumab	Placebo	545	Recurrence-free survival	NCT03383458
KEYNOTE-937	Pembrolizumab	Placebo	950	Recurrence-free survival/overall survival	NCT03867084
SHR-1210-III-325	Camrelizumab + rivoceranib	Active surveillance	687	Recurrence-free survival	NCT04639180

TACE, transarterial chemoembolization.

### Second- or later-line treatment

Molecular-targeted agents developed for second-line therapy include regorafenib, ramucirumab, and cabozantinib (Fig. 1), all of which were developed as agents for second- or later-line treatment after failure of first-line treatment with Sora.

### Regorafenib

Regorafenib is a multi-kinase inhibitor that blocks VEGFR1–3, TIE-2, PDGFRαβ, FGFR, KIT, RET, RAF-1, BRAF, etc. A phase III study comparing this drug with placebo in patients showing radiological progression during Sora treatment, even if they could tolerate Sora (RESORCE trial) [6], showed significantly better survival, progression-free survival, and time-to-progression in the regorafenib arm.

### Ramucirumab

Ramucirumab is a human anti-VEGFR-2 monoclonal antibody that is administered by intravenous injection every 2 weeks. A global phase III trial of ramucirumab versus placebo conducted in patients with unresectable HCC who were refractory to Sora or could not tolerate the drug, with serum AFP levels of ≥400 ng/mL (REACH-2) [7], showed significantly better overall survival and progression-free survival in the ramucirumab arm.

### Cabozantinib

Cabozantinib is a multi-kinase inhibitor that blocks VEGFR, MET, RET, AXL, KIT, and TIE-2. A global phase III trial conducted to compare the usefulness of this drug over placebo in patients receiving second- or later-line treatment for disease progression after at least one systemic therapeutic regimen including Sora (CELESTIAL) [8] demonstrated significantly better overall survival and progression-free survival (both primary endpoints) in the cabozantinib arm.

### Selection of systemic therapy for second- or later-line therapy

There is no consensus on the most appropriate systemic therapy after first-line therapy because no large-scale phase III trials have been conducted for second-line therapy after first-line therapy with Atezo + Bev or Durva + Treme. In Japan, in addition to regorafenib, ramucirumab, and cabozantinib, which have been approved for second- or later-line treatment, other systemic therapeutic agents not used in first-line therapy are considered as valid options for second- or later-line therapy; according to the Japanese Clinical Practice Guideline for Liver Cancer (Table 3), any agent not used in first-line therapy can be selected. For example, if Atezo + Bev is selected as first-line treatment, Len or Durva + Treme can be a selected for second-line treatment. On the other hand, in other countries, the number of drugs available is still limited and the treatments approved for first-line treatment cannot be used for second- or later-line treatments, which is a major difference between Japan and other countries. However, there are numerous critical issues that remain to be clarified, including the efficacy and safety of systemic regimens after Atezo + Bev or Durva + Treme. In addition, there is also a big difference in cost of drug. The treatment cost per first 4 weeks as of January 2025 was 636 451 JPY in regorafenib, 724 064 JPY in ramucirumab assuming an average weight of patients of 60 kg, and 625 324 JPY in cabozantinib. In comparison, the cost per first cycle of 4 weeks was 287 422 JPY in Len and 366 273 JPY in Sora. The cost of Atezo + Bev is not much different from that of molecular-targeted agents, but the cost of Durva + Treme is higher. We should consider the cost of the treatment when selecting the most appropriate treatment. Real-world data from the PRISM (UMIN000040488) and HERITAGE (UMIN000046567) trials may shed light on which regimens should be selected in real-world clinical practice.

### Other phase III trials of second- or later-line therapy that have reported favorable results

A phase III trial conducted only in China has reported the efficacy of apatinib (same agent as rivoceranib) [20], which is being developed as a component of a combination regimen with camrelizumab for first-line therapy. However, no other global trials have reported the efficacy of any other regimen(s).

### Ongoing clinical trials of second-line therapy

A pivotal phase III trial is underway to evaluate the usefulness of adding Atezo, an anti-PD-L1 antibody, to Sora/Len alone, which is often selected as second-line treatment after Atezo + Bev (IMbrave251) (Table 4). The study subjects are patients showing disease progression after Atezo + Bev, and the primary endpoint is the overall survival. A randomized phase II trial of combined therapy with livmoniplimab, which is a humanized monoclonal antibody directed against the transforming growth factor-beta activator and glycoprotein A repetitions predominant, plus budigalimab, a humanized IgG1 monoclonal antibody targeting PD-1 versus Sora or Len for HCC patients showing disease progression after first-line immune checkpoint inhibitor–based therapy, is ongoing. Thus, development of newer agents with different mechanisms of action from those of previously established agents as also of new immunotherapies, such as CAR-T therapy, is underway.

## Systemic therapy for intermediate-stage HCC

Intermediate-stage HCC is defined as multinodular HCC in patients with a performance status of 0, but without evidence of vascular invasion or extrahepatic metastasis [9]. For this stage of the disease, transarterial chemoembolization (TACE) is generally recommended as the treatment of first choice [9] (Table 1). Systemic therapies are used alone or in combination with TACE for intermediate-stage HCC. In this stage, progression-free survival is often selected as a surrogate endpoint of overall survival. It is useful for early and appropriate evaluation of the efficacy of regimens, but it is difficult to judge whether the treatment would actually contribute to prolonging the overall survival.

### Systemic therapy alone

Systemic therapy alone for intermediate-stage HCC is generally only used for patients who are refractory to TACE (TACE-refractory) (Table 5A) or are not suitable candidates for TACE (TACE-unsuitable) (Table 5B). The TACE-refractory status is associated with ineffectiveness of further TACE procedures [21], and the TACE-unsuitable status is determined based on the presence of multiple or large tumors that cannot be expected to be controlled by TACE [22]. For patients with a TACE-refractory status (Table 5A), previous retrospective studies [23,24] and prospective observational trials, including the OPTIMIS trial [25], have shown that switching to systemic therapy at the time of determination of TACE refractoriness was associated with a better time-to-progression and overall survival. Thus, there is some consensus on the treatment of TACE-refractory HCC patients.

**Table 5:** Definition of TACE refractoriness and consensus statement for TACE-unsuitable

**A. Definition of TACE refractoriness**	
(1) Intrahepatic lesion(s)	
	i. Two or more consecutive insufficient responses of the treated tumors (viable lesion >50%) even after changing the chemotherapeutic agents and/or reanalysis of the feeding artery on response evaluation CT/MRI obtained 1–3 months after having adequately performed selective TACE ii. Two or more consecutive progressions in the liver (tumor number increases as compared to tumor number before the previous TACE procedure) even after having changed the chemotherapeutic agents and/or reanalysis of the feeding artery seen on the response evaluation CT/MRI at 1–3 months after having adequately performed selective TACE
(2) Continuous elevation of tumor markers immediately after TACE even though slight transient decrease is observed	
(3) Appearance of vascular invasion	
(4) Appearance of extrahepatic spread	
**B. Consensus statement for TACE-unsuitable**	
Each one of the following 3 clinical conditions that prevent a survival benefit from TACE or conditions that TACE is even harmful:	
(i) Unlikely to respond to TACE: Confluent multinodular type, massive or infiltrative type, simple nodular type with extranodular growth, poorly differentiated type, intrahepatic multiple disseminated nodules, or sarcomatous changes after TACE	
(ii) Likely to develop TACE failure/refractoriness: Up-to-7 criteria out nodules	
(iii) Likely to become Child–Pugh B or C after TACE: Up-to-7 criteria out nodules (especially, bilobar multifocal HCC) mALBI grade 2b	

However, the TACE-unsuitable status (Table 5B) is yet to be fully validated. A multicenter phase II trial of Atezo + Bev (REPLACEMENT) conducted in TACE-unsuitable HCC patients [26] revealed some clinical benefits of the treatment in TACE-unsuitable HCC patients (objective response rate, 45.9%; median progression-free survival, 9.1 months); however, few clinical trials targeting TACE-unsuitable HCC patients have been conducted. In addition, interpretation of the criteria for determination of a TACE-unsuitable status varies among oncologists, hepatologists, and interventional radiologists, and the most appropriate tumor size, tumor number, and hepatic reserve for defining the TACE-unsuitable status are often discussed. With the advent of effective systemic therapies such as Atezo + Bev and Durva + Treme, systemic therapy is actively selected, especially in Japan, for patients in whom disease control by locoregional therapies, such as TACE, is judged as being difficult. The proportion of patients with intermediate-stage HCC in pivotal trials of systemic therapies (IMbrave150, 15.0%; HIMALAYA, 19.6%) was 10%–20% of the overall study population, but 40%–60% of the Japanese population (IMbrave150, 43.0%; HIMALAYA, 58.8%). One of the reasons for this is the difference in the insurance approval and reimbursement statuses between Japan and other countries. As mentioned above, all eight regimens are available for second- or later-line treatment in Japan, while the number of drugs available is still limited in other countries, especially for later-line treatments. Therefore, in Japan, the indication of systemic therapy is already expanding to intermediate-stage HCC.

Patients who are not suitable candidates for local therapy are initiated on systemic therapy, and if this proves successful, they are switched to any local therapies that are available, a strategy which yields favorable therapeutic outcomes. This strategy is attracting attention as conversion therapy. Recently, conversion to local therapies, such as resection, radiofrequency ablation (RFA), and TACE (curative conversion) has been considered for patients showing good outcomes of systemic therapy. This treatment strategy aimed at conversion in patients treated with Atezo + Bev is called ABC-Conversion [27]. Among 110 consecutive patients with Child–Pugh A liver disease who were treated with Atezo + Bev as first-line treatment for unresectable and TACE-unsuitable intermediate-stage HCC, clinical or pathological complete response and curative conversion were achieved in 38 patients (35%) and 35 patients (32%), respectively, by resection, ablation, or selective TACE. Among the 38 patients in whom a complete response was achieved, a drug-free status was eventually achieved in 25 patients [24]. Conversion to surgery in patients receiving treatment with Len alone was also prospectively investigated (LENS-HCC) [28]. A total of 49 patients, mainly those with vascular invasion (65%) and/or extrahepatic metastasis (15%), were enrolled, and the resection rate, the primary endpoint, was 67.4% (33/49); the 1-year survival rate (75.9%) was also favorable. Systemic therapy is generally continued for as long as it is effective, but should an effective local therapy become available, the patients are promptly switched to local therapy. This concept is based on the extensive experience with local therapies such as liver resection, local ablative therapy, and TACE in Japan.

### TACE plus systemic therapy

TACE is primarily a palliative treatment, although selective TACE is sometimes curative. The therapeutic effect of TACE alone is limited, so that the development of TACE combined with systemic therapy has been actively pursued. The TACTICS trial [29] comparing TACE plus Sora with TACE alone showed significantly better progression-free survival in the combination treatment group than in the TACE-alone group (median progression-free survival: TACE plus Sora 25.2 months vs. TACE alone 13.5 months; hazard ratio, 0.59; 95% CI, 0.41–0.87; *P* = .006). Although there was no statistically significant difference in the overall survival (median overall survival: TACE plus Sora 36.2 months vs. TACE alone 30.8 months; hazard ratio, 0.861; 95% CI, 0.607–1.223; *P* = .40), a median survival difference of 5.4 months was observed, which was interpreted as a clinically meaningful difference. In addition, phase II trials of TACE plus Len (TACTICS-L) [30] and TACE plus Nivo (IMMUTACE) [31] have shown favorable antitumor efficacies of both of these treatments, with the best response rates of 88.7% and 71.4%, respectively, and are considered as promising treatment regimens for intermediate-stage HCC.

TACE plus Durva + Bev was demonstrated to show statistically significantly greater clinical improvement and progression-free survival as compared with TACE plus placebo (median 15.0 months vs. 8.2 months; hazard ratio, 0.77; 95% CI, 0.61–0.98; *P* = .032) (EMERALD-1) [32]. As the overall survival was not shown in this presentation, mature overall survival results are still awaited. More recently, a significantly better progression-free survival was demonstrated for TACE plus pembrolizumab + Len (median, 14.6 months) than for TACE + placebo (median, 10.0 months) (hazard ratio 0.66; 95% CI, 0.51–0.84; *P* = .002) [33]. In addition, a favorable trend of overall survival (hazard ratio 0.80; 95% CI, 0.57–1.11; *P* = .0867) and a significantly better objective response rate (TACE plus pembrolizumab + Len, 71.3%; TACE + placebo, 49.8%; *P* < .0001) were also reported for TACE plus pembrolizumab + Len. Although no results for overall survival with TACE plus Durva + Bev were reported from the EMERALD-1 trial, a favorable trend of overall survival was reported for TACE plus pembrolizumab + Len by the LEAP-012 trial, and it is expected that systemic therapy in combination with TACE will be approved in the future for patients with intermediate-stage HCC.

### Ongoing clinical trials of TACE plus systemic therapy

Currently, several pivotal clinical trials comparing TACE alone with TACE plus placebo as the control group are ongoing (Table 4), including the EMERALD-3 trial comparing control with TACE + Durva + Len and TACE + Durva + Treme, the CheckMate-74W trial comparing control with TACE + Nivo + Ipi, the TACE-3 trial comparing control with TACE + Nivo, and the TALENTACE trial comparing control with TACE + Atezo + Bev. The main agents used are immune checkpoint inhibitors, which are considered as the more likely to activate the immune system when used in combination with TACE, yielding synergistic effects and better antitumor efficacy. Since it is difficult to obtain a significant difference in the overall survival, progression-free survival and time-to-TACE failure are often set as the primary endpoints in these trials. However, an additional favorable trend in the overall survival may be also required to reach consensus as a standard treatment. In clinical practice, we should select systemic therapy based on a holistic assessment of the patient.

## Systemic therapy for early-stage HCC

According to the BCLC staging system [9], patients are classified as having early-stage HCC if they have a single tumor nodule or three or fewer nodules measuring ≤3 cm each. Hepatic resection, liver transplantation, or local ablative therapy is recommended as the treatment of first choice for patients with early-stage HCC (Table 1). These therapies are curative, and systemic therapy has been developed as preoperative and/or postoperative adjuvant therapy. Although no standard perioperative treatment has been established yet, the usefulness of Atezo + Bev [34] and sintilimab [35] as postoperative adjuvant therapy has been demonstrated. In this stage, recurrence-free survival is often selected as a surrogate endpoint of overall survival. It is useful for early and appropriate evaluation of the efficacy of regimens, but it is difficult to judge whether the treatments would actually contribute to prolonging overall survival.

**Table 6:** Oncological criteria of resectability for hepatocellular carcinoma according to Japanese expert consensus 2023

**R: resectable**
Oncological status for which surgery alone may offer clearly better survival outcomes compared to the other treatment: (i) and (ii) and (iii)
(i) Solitary lesion (no size limit) or multiple lesions up to 3 nodules each ≤3 cm
(ii) No macrovascular invasion detected on image (Vp0–1 and Vv0–1 and B0–1)
(iii) No extrahepatic disease
**BR1: borderline resectable 1**
Oncological status for which surgical intervention as a part of multidisciplinary treatment may offer survival benefit: at least one of the following:
(i) Multiple lesions exceeding the criteria of R, but no more than 5 nodules and 5 cm in diameter
(ii) Macrovascular invasion (Vp2–3 or Vv2 or B2–3)
(iii) Localized extrahepatic disease
(e.g. solitary nodal involvement at no. 3, 8, or 12 lymph node/localized peritoneal dissemination/unilateral adrenal metastasis/or oligometastasis to the lung)
**BR2: borderline resectable 2 (initially unsuitable for resection)**
Oncological status for which efficacy of surgery is indeterminate and surgical indication should be carefully determined under the standard multidisciplinary management of HCC: at least one of the following:
(i) Multiple lesions >5 nodules or >5 cm in diameter
(ii) Major vascular invasion (Vp4 or Vv3 or B4)
(iii) Extrahepatic disease not fulfilling the localized factor classified as BR1
Note. Oncological criteria of resectability should be determined independently from technical and/or functional criteria of resectability

Vp0, no tumor thrombus; Vp1, tumor thrombus in the third or lower branch of the portal vein; Vp2, tumor thrombus in the second branch of the portal vein; Vp3, tumor thrombus in the first branch of portal vein; Vp4, tumor thrombus in the main portal trunk or opposite side of the portal branch; Vv0, no tumor thrombus; Vv1, tumor thrombus in a peripheral branch of the hepatic vein; Vv2, tumor thrombus in the main trunk of the right, middle, or left hepatic vein; Vv3, tumor thrombus in the inferior vena cava; B0, no tumor thrombus; B1, tumor thrombus in the third or lower branch of the bile duct; B2, tumor thrombus in the second branch of the bile duct; B3, tumor thrombus in the first branch of bile duct; B4, tumor thrombus in the common hepatic duct.

### Postoperative adjuvant therapy

#### Phase III trial of Atezo + Bev versus active surveillance (IMbrave050)

The results of the IMbrave050 trial in which Atezo + Bev was compared with active surveillance as adjuvant therapy after surgical resection/local ablation for patients of HCC with a high-risk recurrence were reported in 2023 [34]. The primary endpoint of recurrence-free survival was significantly better in the Atezo + Bev group than in the active surveillance group (12-month recurrence-free survival rate: Atezo + Bev 79% vs. active surveillance 68%; hazard ratio, 0.70; 95% CI, 0.54–0.91; *P* = .007). However, there was no difference in the overall survival (hazard ratio, 1.42; 95% CI, 0.80–2.54); furthermore, the Kaplan–Meier curves for recurrence-free survival in the two groups became closer after 2 years, leading some to argue that the treatment could only be considered as being effective for suppressing early recurrence. However, in the first analysis, the median follow-up period (17.4 months) was still short and overall survival events occurred in only 7% of the patients. Recently, the updated results of analysis after a follow-up period of 35.1 months [36] were reported. The median recurrence-free survival was 33.2 months in the Atezo + Bev arm and 36.0 months in the active surveillance arm, with a hazard ratio of 0.90 (95% CI, 0.72–1.12), showing no significant difference. The overall survival was also not favorable, with a hazard ratio of 1.26 (95% CI, 0.85–1.87).

#### Phase II trial of sintilimab versus active surveillance

A randomized phase II trial reported favorable results of adjuvant treatment with the anti-PD-1 antibody, sintilimab, as compared with active surveillance, after curative resection in HCC patients with vascular invasion [35]. The adjuvant sintilimab arm showed significantly better recurrence-free survival (median recurrence-free survival time: sintilimab 27.7 months vs. active surveillance 15.5 months; hazard ratio, 0.534; 95% CI, 0.360–0.792; *P* = .002) and a trend toward better overall survival (2-year overall survival rate: sintilimab 87.9% vs. active surveillance 78.0%; hazard ratio, 0.505; 95% CI, 0.254–1.006).

### Ongoing clinical trials of adjuvant therapy after curative treatment

Some pivotal clinical trials of postoperative adjuvant therapy for early-stage HCC are currently underway (Table 4): the EMERALD-2 trial, evaluating the efficacy of adjuvant Durva + Bev, the CheckMate9DX trial evaluating the efficacy of adjuvant Nivo, the KEYNOTE-937 trial evaluating the efficacy of adjuvant pembrolizumab, and the SHR-1210-III-325 trial evaluating the efficacy of adjuvant camrelizumab + rivoceranib, as compared with placebo or observation in the control group. The results of these trials could be expected to clarify whether a single immune checkpoint inhibitor will be sufficient or a combination of an immune checkpoint inhibitor and a VEGF inhibitor might be necessary to obtain clinical benefit from postoperative adjuvant therapy. Furthermore, it is still not fully understood if recurrence-free survival might be an accurate predictor of the overall survival, and further research is needed to clarify this. Also, we should to select the most appropriate treatment based on a holistic assessment of the patient.

### Preoperative neoadjuvant therapy

In addition, development of preoperative adjuvant therapy is also considered necessary for HCC patients with a high risk for recurrence. Some reports suggesting the effectiveness of preoperative neoadjuvant treatment for early-stage HCC have also been published. In a randomized phase II trial of neoadjuvant Nivo versus Nivo plus ipilimumab for resectable HCC, major pathological response with no recurrence was achieved in 6 of the 27 enrolled patients [37]. In a phase II trial of neoadjuvant anti-PD-1 antibody, cemiplimab has been reported to provide favorable outcomes in patients with resectable HCC [38]. Among 21 enrolled patients with resectable HCC, surgical resection was successfully performed in 20 patients, major pathological response was achieved in 4 patients (20%), and partial response was achieved in 3 patients (15%). Thus, the feasibility of neoadjuvant immunotherapy and some effective outcomes have been demonstrated. These findings lend further support to the rationale for developing neoadjuvant immunotherapy for resectable HCC.

### Borderline resectable HCC

In pancreatic cancer, the concept of “borderline resectable” has become firmly established and neoadjuvant therapy is considered mandatory. The concept of “borderline resectable,” which is defined as technically resectable, but oncologically unresectable, has also been introduced for the case of HCC [39]. The oncological criteria for resectability of HCC have been proposed by the Japanese Expert Consensus in 2023 (Table 6). Based on these, HCC has been classified as resectable, borderline resectable 1 (BR1) and borderline resectable 2 (BR2), and BR1 and BR2 are good candidates for preoperative neoadjuvant treatment. Currently, a multicenter phase II clinical study (RACB study) (UMIN000046634) is underway to investigate the safety and efficacy of multimodality treatment with Atezo + Bev and surgical resection in patients with unresectable HCC, including borderline resectable HCC [40], and a phase II study (LEOPARD-Neo) (jRCTs031230128) is underway to investigate the efficacy of Len plus hepatic arterial infusion chemotherapy with cisplatin for borderline resectable HCC. A multicenter phase II trial [41] showed that Len + hepatic arterial infusion chemotherapy with cisplatin exerted a high tumor shrinkage effect (objective response rate, 64.7% by modified RECIST, and 45.7% by RECIST version 1.1) in patients with unresectable HCC, and it was adopted as neoadjuvant treatment. In the near future, multidisciplinary treatments with preoperative and postoperative adjuvant therapies may become the standard for patients with resectable HCC.

## Conclusions

In the early 2000s, surgical resection, RFA, and TACE were the three major treatment modalities for HCC. However, with the advent of combined immunotherapy regimens, the perspective of systemic therapy for HCC has changed dramatically, with systemic therapy being used as adjuvant therapy after resection/RFA for patients with early-stage (BCLC) HCC, in combination with TACE for patients with intermediate-stage HCC, and as not only first-line, but also second- or later-line therapy for patients with advanced-stage HCC. However, several critical issues still need to be resolved, including how to structure the treatment plan to ensure long-term survival while maintaining the hepatic reserve in patients with HCC. In addition, the concept of conversion therapy after systemic therapy is also emerging, and the significance of multidisciplinary treatment for HCC is increasing. Further novel clinical studies and clinical trials with originality and ingenuity are warranted to achieve better treatment outcomes and prolonged survival in patients with HCC. Finally, it is important to develop a treatment strategy based on a holistic assessment of the patient, also taking into account the cost of the treatment.
